# Down-Regulation of Collagen Hydroxylation in Colorectal Liver Metastasis

**DOI:** 10.3389/fonc.2020.557737

**Published:** 2020-09-30

**Authors:** Nick A. van Huizen, Peter C. Burgers, Joost van Rosmalen, Michail Doukas, Jan N. M. IJzermans, Theo M. Luider

**Affiliations:** ^1^Department of Surgery, Erasmus University Medical Center, Rotterdam, Netherlands; ^2^Department of Neurology, Erasmus University Medical Center, Rotterdam, Netherlands; ^3^Department of Biostatistics, Erasmus University Medical Center, Rotterdam, Netherlands; ^4^Department of Pathology, Erasmus University Medical Center, Rotterdam, Netherlands

**Keywords:** post-translational modification, mass spectrometry, colorectal cancer, colorectal liver metastasis, collagen, hydroxyproline

## Abstract

Collagen is significantly upregulated in colorectal liver metastasis (CRLM) compared to liver tissue. Expression levels of specific collagen types in CRLM resemble those in colorectal cancer (CRC) and colon tissue. We investigated whether the collagen hydroxylation pattern from the primary tumor also migrates with the metastatic tumor. The degree of collagen alpha-1(I) hydroxylation in colon, CRC, liver, and CRLM tissue of the same individuals (*n* = 14) was studied with mass spectrometry. The degree of hydroxylation was investigated in 36 collagen alpha-1(I) peptides, covering 54% of the triple helical region. The degree of hydroxylation in liver tissue was similar to that in colon tissue. The overall degree of hydroxylation was significantly lower (9 ± 14%) in CRC tissue and also significantly lower (12 ± 22%) in CRLM tissue compared to colon. Furthermore, eleven peptides with a specific number of hydroxylations are significantly different between CRLM and liver tissue; these peptides could be candidates for the detection of CRLM. For one of these eleven peptides, a matching naturally occurring peptide in urine has been identified as being significantly different between patients suffering from CRLM and healthy controls. The hydroxylation pattern in CRLM resembles partly the pattern in liver, primary colorectal cancer and colon.

## Introduction

Collagen is a family of proteins that form triple helical structures. A triple helix is formed by three protein subunits called alpha-chains. The specific part of an alpha-chain involved in triple helix formation consists of a repeating unit [-Gly-Xaa-Yaa-]_n_ where Xaa and Yaa can be different amino acids, frequently proline and lysine (1). Proline and lysine can be enzymatically modified prior to triple helix formation. Proline is hydroxylated into hydroxyproline (Hyp); two different Hyp isomers exist in collagen, namely 4-hydroxyproline (4Hyp) and—more rarely—3-hydroxyproline (3Hyp). Normally, 4Hyp at the Yaa-position is formed by 4-prolyl hydroxylase, which requires Gly-Xaa-Pro as substrate (2). 4-hydroxyproline rarely occurs at the Xaa positions (4xHyp), neither the enzyme, nor the substrate required for the formation of 4xHyp is known (3). 3Hyp is formed by 3-prolyl hydroxylase, which requires Gly-Pro-4Hyp as substrate (4). 3Hyp and 4Hyp are involved in triple helix stability and fiber formation, but their specific functions are still not fully understood (5–7). The function of 4xHyp remains unknown (3). Lysine can be modified into 5-hydroxylysine (5Hyl) by lysyl hydroxylases (8). In addition, 5Hyl can be further glycosylated (9) and is involved in collagen cross-linking (10). After triple helix formation, collagen triple helices are excreted from the cell via the Golgi apparatus into the extracellular matrix. In the extracellular matrix, triple helices form a variety of supramolecular structures (11).

Triple helix folding is reversible; unfolding occurs when the melting temperature (T_m_) is exceeded (12). The T_m_ is influenced by the physical properties of the amino acids at the Xaa and Yaa positions (1) and post-translational modifications (PTMs). In short model peptides, hydroxylation of proline into 4Hyp increased the T_m_ up to 6°C, while conversion into 3Hyp decreased the T_m_ (6). The effect of 5Hyl on the T_m_ remains unknown. Leikina et al. (13) demonstrate that the T_m_ of collagen type I in lung is just below body temperature, in addition, only folded triple helices are present below 30°C. If the number of PTM's decreases, then this would result in collagen with a lower Tm. Collagen with a lower Tm will unfold/melt before it can be built into fibers and will be degraded by the body.

The 4Hyp formation rate is influenced by the amino acid at the Xaa position (14), the temperature at which collagen-producing cells are cultured (15), and the amount of oxygen present (16). Collagen produced under hypoxic conditions has ~1.7% less Hyp and almost 10% less Hyl (16). Under hypoxic conditions, proline hydroxylation is the rate limiting step of collagen formation, indicating that a minimum number of PTMs is required to form stable collagen. Because collagen hydroxylation is influenced by many parameters, we hypothesize that the hydroxylation pattern varies strongly between different tissues and within a particular tissue. In agreement with this, Montgomery et al. (17) described that the degree of hydroxylation in collagen alpha-1(I) of ten breast cancer patients varied between 0 and 90%, depending on the proline position.

Collagen levels in tumor tissue are dysregulated in comparison to healthy tissue (18). In colorectal liver metastasis (CRLM), collagen levels are, in general, significantly upregulated in comparison to adjacent normal liver tissue (19, 20). Collagen type 10, 12, 14, and 15 expression levels have most likely migrated from the healthy colon tissue, via the primary tumor (colorectal cancer; CRC), to the metastasis. The migration of colon-specific protein expression into CRLM is not limited to collagen; other proteins, e.g., CDH17, KRT20, CEACAM5, GPA33, MUC13, and PPP1R1B/DARPP32 show a similar pattern, although many colon-specific proteins are down-regulated too (20).

In this study, we have investigated the hydroxylation pattern, taking into account the hydroxylation of proline and lysine in collagen alpha-1(I), to better understand the changes in collagen caused by CRLM. We hypothesize that the hydroxylation pattern co-migrates from colon tissue, via the primary tumor, toward the metastasis similarly as a number of colon-specific proteins and specific collagen types. In addition, it was investigated if studying the collagen hydroxylation pattern can aid in the finding of biomarkers to further improve the diagnoses of CRLM based on urine (21). Collagen hydroxylation was studied with bottom-up mass spectrometry in colon, CRC, CRLM, and liver tissue obtained from the same individual.

## Materials and Methods

### Sample Selection

Bottom-up proteomics data of matching normal liver tissue and colorectal liver metastasis (CRLM) tissue of 30 patients were retrieved from the PRIDE Archive (PXD008383). Normal liver tissue was taken at least 1 cm away from the tumor (20). We could collect matching healthy adjacent colon tissue and CRC tissue from 14 out of these 30 patients. The tissue material had been collected during curative surgery. Morphology differences indicated that the CRC tissues contained adjacent normal tissue, and that one colon tissue contained tumorous tissue. None of the CRLM tissues contained non-tumor material, and none of the liver tissues contained non-normal tissue. This study was approved by the Erasmus MC ethics review board (MEC-2007-088) and we have worked according to the declaration of Helsinki. An experienced pathologist (MD) has reviewed the sections.

### Sample Preparation and Data Processing

All tissues were prepared, measured, and data processed as described by van Huizen et al. (20). In short, formalin cross-links were removed by incubation with 1M TRIS pH 8, followed by cysteine reduction and alkylation. The proteins in the tissue were cleaved into peptides by overnight incubation with trypsin, 0.6 μg of trypsin was added. Trypsin cleaves after lysine and arginine except if a proline is present after these two amino acids. Peptides were identified with nanoLC-MS/MS measurements on an Orbitrap Fusion Tribrid Mass Spectrometer (Thermo Fischer Scientific, San Jose, CA, USA). MGF peak list files were extracted from raw files by ProteoWizard (v3.0.9166) and searched with Mascot (v2.3.2, Matrix Science Inc., London, UK) and the UniProt/Swiss-prot Human database (20,194 entries, v2015_11). Hydroxylations (+16 Da) of proline, lysine, and methionine were included as variable modifications and carbamidomethylation (+57 Da) of cysteine as fixed modification. A maximum of 4 missed cleavages was allowed. Mascot search results were further analyzed in Scaffold (v4.6.2, Portland, OR, USA). In Scaffold, protein confidence levels were set to a 1% false discovery rate (FDR), at least 2 peptides per protein, and a 1% FDR at the peptide level. FDRs were estimated by inclusion of a decoy database search generated by Mascot. A Peptide Report exported from Scaffold was used for data analysis. The peptide report regarding liver and CRLM tissue has been published (20), the peptide report regarding colon and CRC tissue is included in the Supplementary S1. Mass spectrometry data was made publicly accessible via the PRIDE archive, accession number: PXD015015.

### Peptide Panel Selection

Collagen alpha-1(I) peptides were selected on the following criteria: (1) does not contain methionine or cysteine; (2) contains at least one proline or lysine moiety; and (3) a unique number of hydroxylations, having been identified in 20 or more liver or CRLM tissues, and in 3 or more colon and CRC tissues. The latter sub criterion was applied because the statistical formulas used require a sample size of 3 or larger.

### Principal Components Analysis

Principal component analysis (PCA) was performed in R (v3.6.0.) (22); the R-script can be found in the Supplementary S2. A table was created containing for every sample a value for the absence (value 0) or presence (value 1) for each peptide with a unique number of hydroxylations. Zero to 10 hydroxylations were taken into consideration; where 10 is the maximum number of hydroxylations observed for the peptides in the peptide panel. The PCA was calculated with the “prcomp” function. If two or more tissue types were compared, then a 2D-PCA-plot was made with the package “factoextra.” 3D-PCA-plots were made by exporting the coordinates of the first three PCA dimensions, and plotting with “scatter3d.” For assessment of the data, 2D-PCA-plots were made by exporting the PCA coordinates, and plotting with “ggplot2.”

### Hydroxylation Analysis

**Figure 1:**
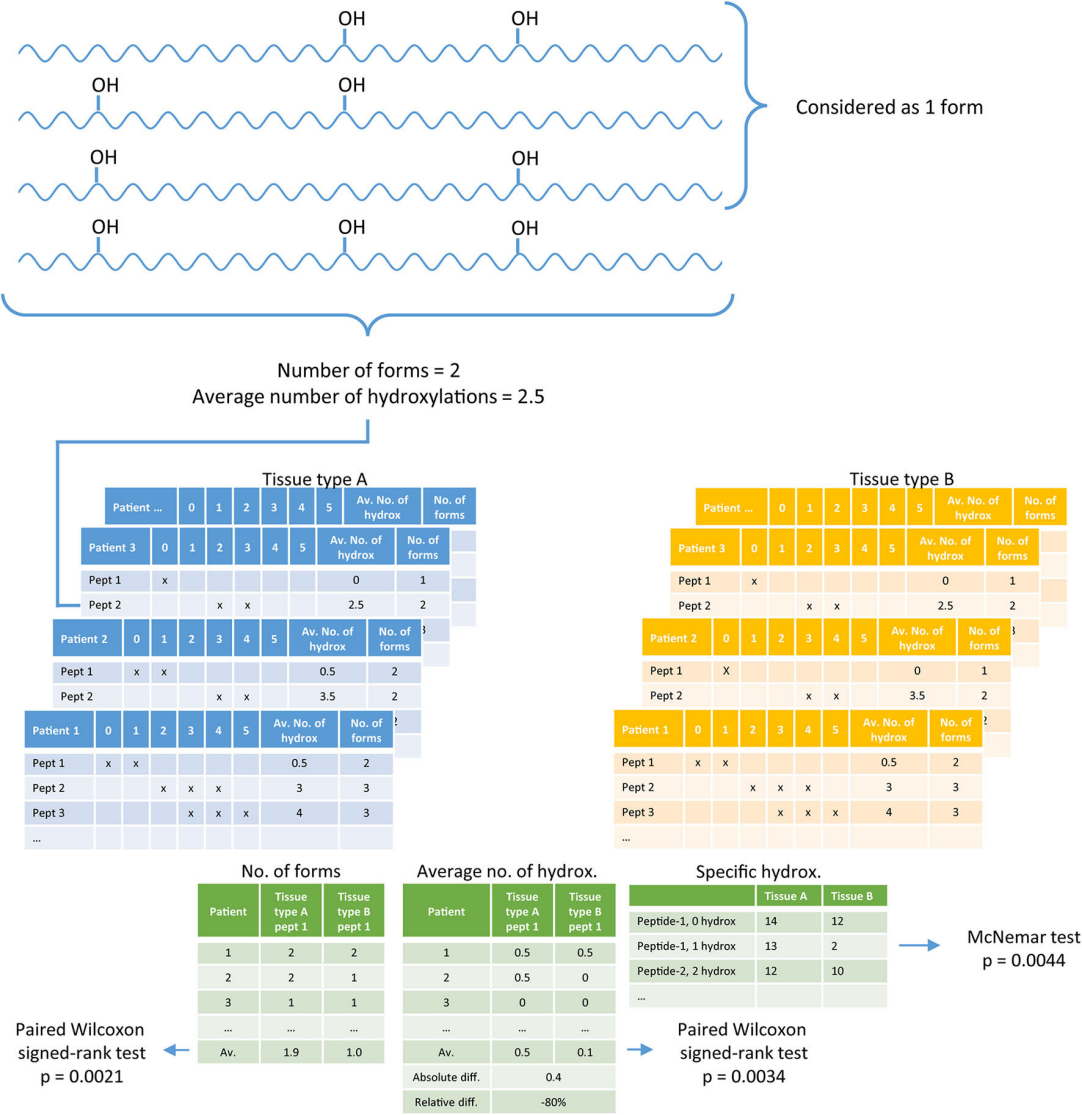
A diagram explaining the data analysis. Per tissue, per patient the number of forms present in collagen peptides, and the average number of hydroxylations per peptide is determined (blue and yellow tables). Per peptide, per patient these two values are compared between two tissue types, left and middle green table. In addition, every form (specific hydroxylation) is separately compared between two tissue types based on the number of patients in which the specific form is identified, right green table.

Statistical analysis was performed in R (v3.6.0.) (22), the R-script can be found in the Supplementary S3. In the digested tissue, collagen peptides are present with different numbers of hydroxylations per peptide. A difficulty with collagen analysis is the identification of the exact location of a hydroxylation. To avoid the identification of the exact hydroxylation position, we used the number of hydroxylations per peptide obtained by the parent mass. Per tissue type (*n* = 4), per patient (*n* = 14 and *n* = 30), and per peptide from the peptide panel (*n* = 36), we checked the presence of 0 to 10 hydroxylations taking into account both hydroxyproline and hydroxylysine, which generated a three-dimensional data matrix. Per peptide a list was generated containing the numbers of different forms observed, compared between two tissue types. Testing for differences between tissue types was performed with a paired Wilcoxon signed-rank test. Per tissue a list was generated containing the average number of hydroxylations per peptide, which was compared between two tissue types with a paired Wilcoxon signed-rank test. If a peptide was not identified in tissue from a particular patient, no average could be calculated, then this patient was removed from analysis of the average number of hydroxylations. Besides the overall differences between the number of different forms observed, and the average number of hydroxylations per peptide, we also studied in more detail if there are peptides with a specific number of hydroxylations that is different between different tissues. The difference in a specific hydroxylation form between two tissue types was assessed with the McNemar test. Two-sided *p-*values below 0.05 were considered as significant. Figure 1 shows an example of the set-up of the data.

### Data Assessment

The quality of the data was assessed prior to the analysis of collagen hydroxylation. A bias in the data based on patient characteristics (age and gender) was investigated by making 2D-PCA plots per tissue type. For cancerous tissue also the location of the primary tumor was taken into account. Furthermore, colon and CRC tissue contained non-normal and non-tumor tissue, respectively. Therefore, an additional 2D-PCA-plot was made containing colon and CRC tissue to assess the presence of a bias based on the percentage of non-normal and non-tumor tissue.

Permutation testing (i.e., randomly reassigning samples to the groups) was used to estimate background in the distribution of the number of forms, the average number of hydroxylations and specific hydroxylation forms between different tissue types. For each peptide, the distribution of the number of forms, the average number of hydroxylations and specific hydroxylation forms was compared between liver and CRLM (both *n* = 30 and *n* = 14) and between other pairs of organs (*n* = 14) using a paired Wilcoxon signed-rank test with a critical *p-*value of 0.05. To demonstrate the significant differences present between the unpermitted data (i.e., the true data), the permutation results were used as a test statistic. The critical value of the permutation test was calculated as the 95th percentile of the number of peptides with a significant difference when samples were permutated. The significance of the number of differences found in the unpermitted data was expressed as a *p-*value. This *p-*value was calculated as the proportion of the permutations where the number of peptides with a significant difference was at least as large as in the unpermitted data. The test used 1,000 random permutations.

Presence of linear relation between the number of forms and the average number of hydroxylations was tested per tissue with the Pearson correlation test. A linear relation was considered significant if the *p-*value was below 0.05/4 = 0.0125 (Bonferroni correction for multiple testing, *n* = 4).

### Analysis of the Hydroxylation Variation

The hydroxylation pattern was compared between: colon and liver, colon and CRC, CRC, and CRLM, colon and CRLM, and liver and CRLM. The difference in the degree of hydroxylation was analyzed in different directions: (1) 2D- and 3D-PCA-plots; (2) the number of forms observed; (3) detailed analyzes of the average number of hydroxylations; and (4) analyses of a specific hydroxylation form. Detailed information regarding the average number of hydroxylations was obtained by: plotting the average number of hydroxylations per peptide; the absolute difference in the average number of hydroxylations per peptide of CRC, CRLM, and liver tissue with respect to colon tissue (tissue—colon tissue); and the relative difference with respect to colon tissue ([tissue/colon −1]^*^100%). Differences between all groups were tested with independent samples *t-*tests. For the average number of hydroxylations per peptide, *p-*values below 0.05/6 (Bonferroni correction, 6 comparisons), and for the relative differences, *p-*values below 0.05/3 (Bonferroni correction, 3 comparisons) were considered significant. The average of the relative difference was compared to zero with a one-sample *t-*test; *p-*values below 0.05/3 (Bonferroni correction, 3 comparisons) were considered as significant.

### Urine Analysis

The value of the significant different peptides identified in section Analysis of the Hydroxylation Variation as a biomarker has been investigated by reanalyzing the published data (PXD013533) by van Huizen et al. (21). The bottom-up mass spectrometry data was processed as described by van Huizen et al., whereby we considered a peptide as significant when a *p-*value was below 0.05.

## Results

### Patient Characteristics

An overview of all patient characteristics is present in Supplementary S4. CRC patients (*n* = 14) had a median age of 64 years [interquartile range [IQR] 57–73] and were mostly male (64%). The CRC tumor had a median size of 4.0 cm (IQR 3.3–4.8), being moderately differentiated adenocarcinomas. CRC was located in rectum (64%, 9 out of 14) or sigmoid (36%, 5 out of 14). Liver and CRLM tissue were not collected simultaneously with colon and CRC tissue. The CRLM patients had a median age of 65 years (IQR 59–73) and a median of 2 (maximum 7) tumors with a median size of 2.3 cm (IQR 1.2–3.5), being moderately differentiated adenocarcinomas.

Prior to surgical removal of CRC or CRLM some patients have received pretreatment in the form of chemotherapy (CRC = 0, CRLM = 6), or a combination of chemotherapy and radiotherapy (CRC = 2, CRLM = 1). In Supplementary S5, we have investigated if a clustering based on the pretreatment is present in a 2D-PCA-plot, and we did not observe a correlation.

In some patients molecular diagnostics has been performed on the CRC (*n* = 7) or CRLM (*n* = 4). Only in three of the seven CRC tumor tissues on which molecular diagnostics has been performed are included in this research (*n* = 3). In Supplementary S5, we have investigated if a clustering in 2D-PCA-plot based on the results of the molecular diagnostics is present, and we did not observe a correlation.

### Peptide Selection

All tissue samples were digested with trypsin and measured via a proteomics bottom-up approach. Mass spectra were searched against the Uniprot/Swissprot human protein database, having hydroxylation of proline and lysine as a variable modification. The database search resulted in the significant identification of, among others, collagen alpha-1(I) peptides.

The number of identified collagen alpha-1(I) peptides with unique numbers of hydroxylation differed per tissue type (ANOVA *p* < 0.001), only colon and CRC were not significantly different (Bonferroni *post-hoc* testing *p* > 0.05) and all other comparisons were significantly different (Bonferroni *post-hoc* testing *p* < 0.0001). The following average numbers of peptides with unique number of hydroxylations were identified in liver 70 (*n* = 30, SD=17), CRLM 111 (*n* = 30, SD = 23), colon 162 (*n* = 14, SD = 10), and CRC 168 (*n* = 14, SD = 28). A total of 113 different peptides with a unique number of hydroxylations were identified, which did neither contain methionine nor cysteine. From these 113 peptides a panel of peptides was selected based on the following selection criteria: (1) does not contain methionine or cysteine; (2) contains at least one proline or lysine moiety; (3) a specific number of hydroxylations in a specific collagen peptide ought to be identified in 20 or more liver or CRLM tissues, and in 3 or more colon and CRC tissues. Thirty-six peptides fulfilled all selection criteria These 36 peptides covered 54% of the triple helical region of collagen alpha-1(I), Supplementary S5. They are ranked corresponding to their occurrence in the primary structure of collagen, and will be referred in the text as, for example, peptide 1. Supplementary S5 also contains per tissue a table specifying the number of hydroxylations on the peptides present.

### Data Assessment

Prior to analysis of the collagen hydroxylation pattern, the data was assessed to investigate if any bias is present in the data that would indicate that the degree of hydroxylation is related to a patient characteristic. Data assessment was performed by making 2D-PCA-plots and a permutation test.

In the constructed 2D-PCA-plots we investigated if a relation between the collagen hydroxylation and patient characteristics was present (Supplementary S5). The 2D-PCA-plot is based on the absence or presence of a peptide with a specific number of hydroxylations. The patient characteristics are visualized by specific shapes, colors, and different sizes of the data points. The axes of the 2D-PCA-plots describe the first and second dimension variability of the first two principal components (PC).

The degree of collagen hydroxylation in CRLM and CRC tissue showed no clustering based on patient age, gender, or location of the (primary) tumor. Likewise, the degree of hydroxylation in liver and colon tissue showed, based on 2D-PCA-plots, no clustering based on patient age or gender. All CRC tissues contained to some extent normal tissue, and just one colon tissue contained some tumorous tissue. Nevertheless, colon and CRC samples still clustered based on the tissue type. The 2D-PCA-plots are available in the Supplementary S5.

**Figure 2:**
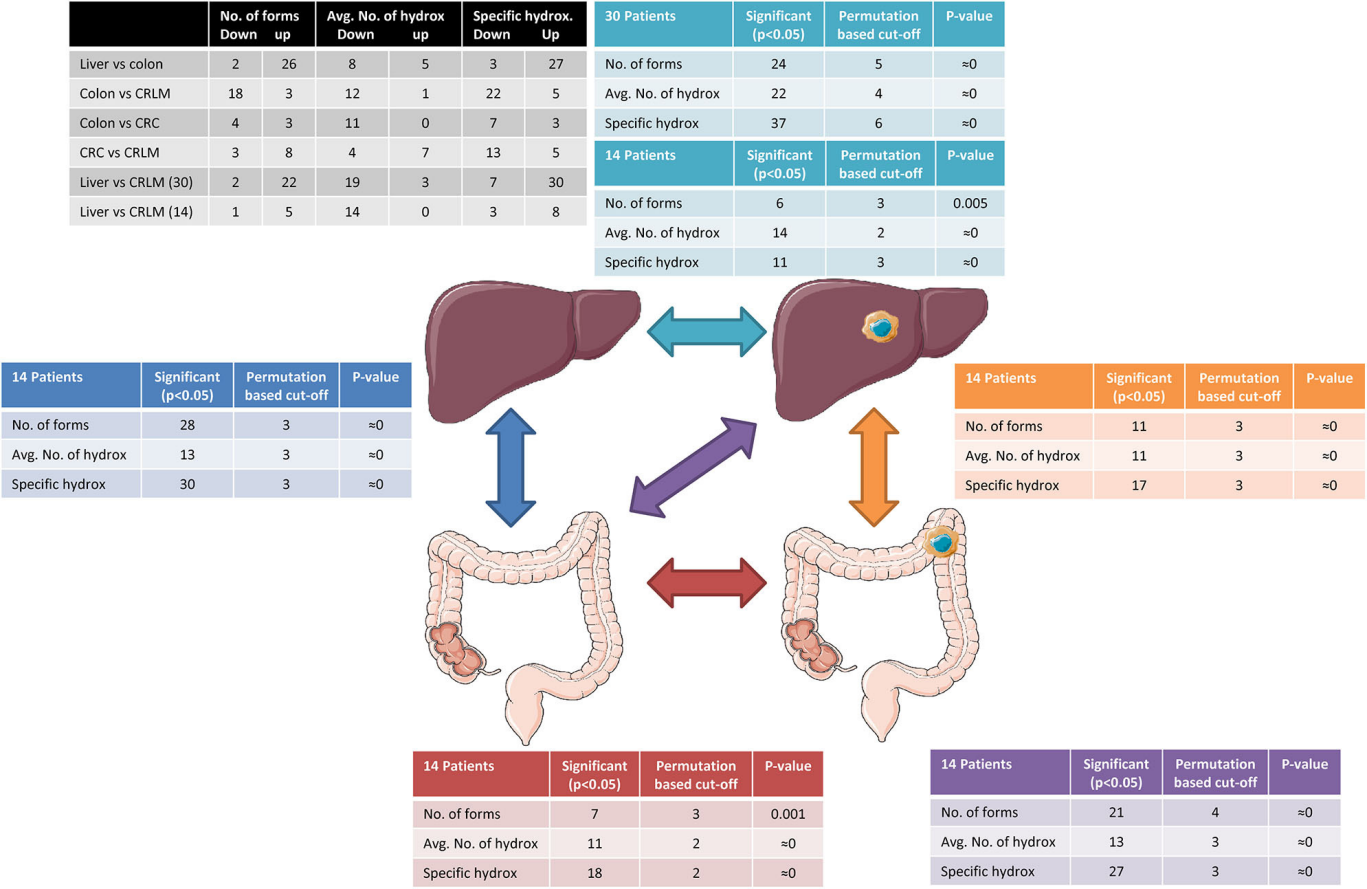
Tissue comparison analysis. The black table shows the number of peptides down- or upregulation regarding the differences in the number of forms, the average number of hydroxylations, and specific hydroxylations. The arrows indicate the comparisons made and tables surrounding the tissue cartoons having the same color belong together. Reprinted (adapted) with permission from (21). Copyright (2019) American Chemical Society.

Permutation testing results are presented in Figure 2. The permutation test indicates that, differences between two tissue types are significant (*p* < 0.05) if more than 4 differences in the number of forms, 3 differences between the average number of hydroxylations per peptide, or 4 specific hydroxylation forms are found. In all comparisons more significant differences were present than expected by permutation testing. The within tissue variation in liver and CRLM tissue is similar to the number of differences observed by chance between the different tissue comparisons, indicating that the differences between tissue are significantly larger than within a tissue.

### Tissue Comparison

**Figure 3:**
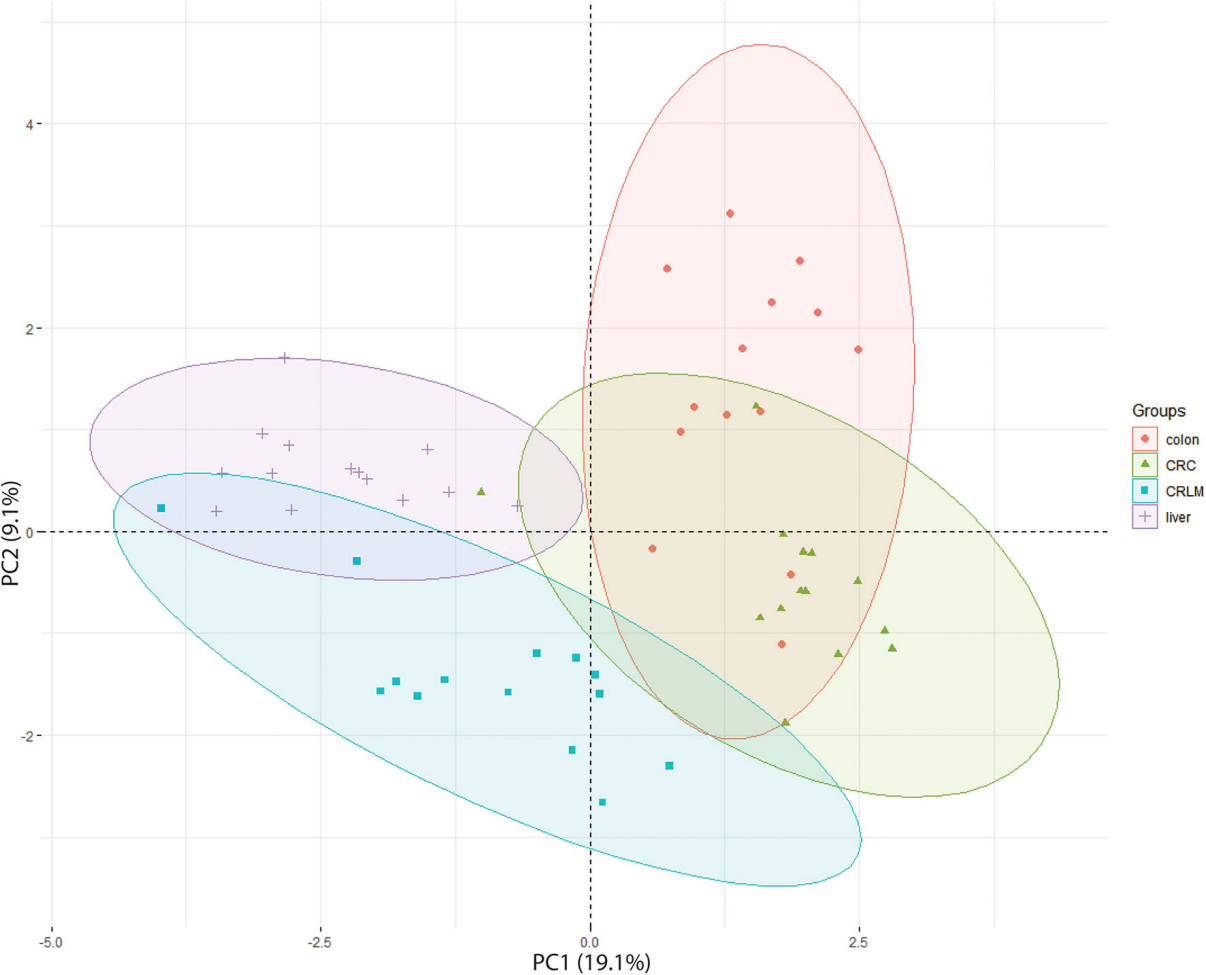
2D-PCA-plot showing the separation of all 4 tissue types based on their degree of hydroxylation. The x-axis is the first principal component (PC1) of the PCA, which explains 19.1% of the variation. The y-axis is the second principal component (PC2) of the PCA, which explains 9.1% of the variation.

In the 2D-PCA-plot shown in Figure 3, liver, CRLM, and colon are separated based on their hydroxylation pattern. CRC is separated from liver and CRLM tissue, but overlaps with colon tissue. Two exceptions are observed. Patient CRLM-10 is located (coordinates: −3.98, 0.22) in between the liver samples, most likely because only 23 different peptides with a unique number of hydroxylations were identified (liver average = 62). Patient CRC-19 is located (coordinates: −1.01, 0.38) in between the liver samples, because only 69 different peptides with a unique number of hydroxylations were identified (CRC average = 92). A 3D-PCA-plot based on the first three dimensions results in a complete separation between liver, CRLM, and Colon+CRC, while colon and CRC are still partly overlapping. The 3D-PCA-plot is available in the Supplementary S5. The 2D-PCA-plots and 3D-PCA-plots for individual tissue comparisons are also available in the Supplementary S5. For the comparison “liver vs. colon,” there is a complete separation in a 2D-PCA-plot and 3D-PCA-plot. For “colon vs. CRC” there is a partial overlap in the 2D-PCA-plot and 3D-PCA-plot. For “CRC vs. CRLM,” and “liver vs. CRLM” (14 vs. 14) there is a slight overlap in the 2D-PCA-plot and they are fully separated in the 3D-PCA-plot. “Liver vs. CRLM” (30 vs. 30) are partly overlapping in the 2D-PCA-plot, and fully separated in the 3D-PCA-plot.

An overview of the results of the tissue comparisons based on the number of forms, average number of hydroxylations, and specific hydroxylations is presented in Figure 2. A Pearson correlation test was performed to exclude a relation between the number of different forms observed and the average number of hydroxylations per peptide. Significant correlations were not found (p-critical = 0.05/4 = 0.0125, Bonferroni correction); liver: *r* = 0.40, *p* = 0.018 CRLM: *r* = 0.31, *p* = 0.066; colon: *r* = 0.21, *p* = 0.21; CRC: *r* = 0.16, *p* = 0.35.

The number of significant peptides and the direction (down- or upregulated) indicate the similarity between the different tissue types. It is worth noting that colon and lover tissue show the highest difference in the average number of hydroxylations, although it is equally down- and upregulated. While colon and CRC have only 11 significant differences in the average number of hydroxylations, the degree of hydroxylation is strongly downregulated in CRC tissue. Also between CRC and CRLM only 11 significant differences were present in the average number of hydroxylations, however in this case they are more equally down- and upregulated.

To compare the effect of group size, the comparison “liver vs. CRLM” was performed with the patients of whom colon and CRC tissue was collected (14 vs. 14) and with the full data set (30 vs. 30). As expected, in the full data set, more and stronger significant differences were observed, because more samples result in a better powered analysis.

In all tissue comparisons the total number of significant differences with respect to the average number of hydroxylations is similar. However, the comparisons of “liver vs. colon,” and “CRC vs. CRLM” have a similar number of down- and upregulated averages (black table Figure 3), while the comparisons “colon vs. CRC,” “colon vs. CRLM,” and “liver vs. CRLM” have an overall increase in downregulation of the average.

**Figure 4:**
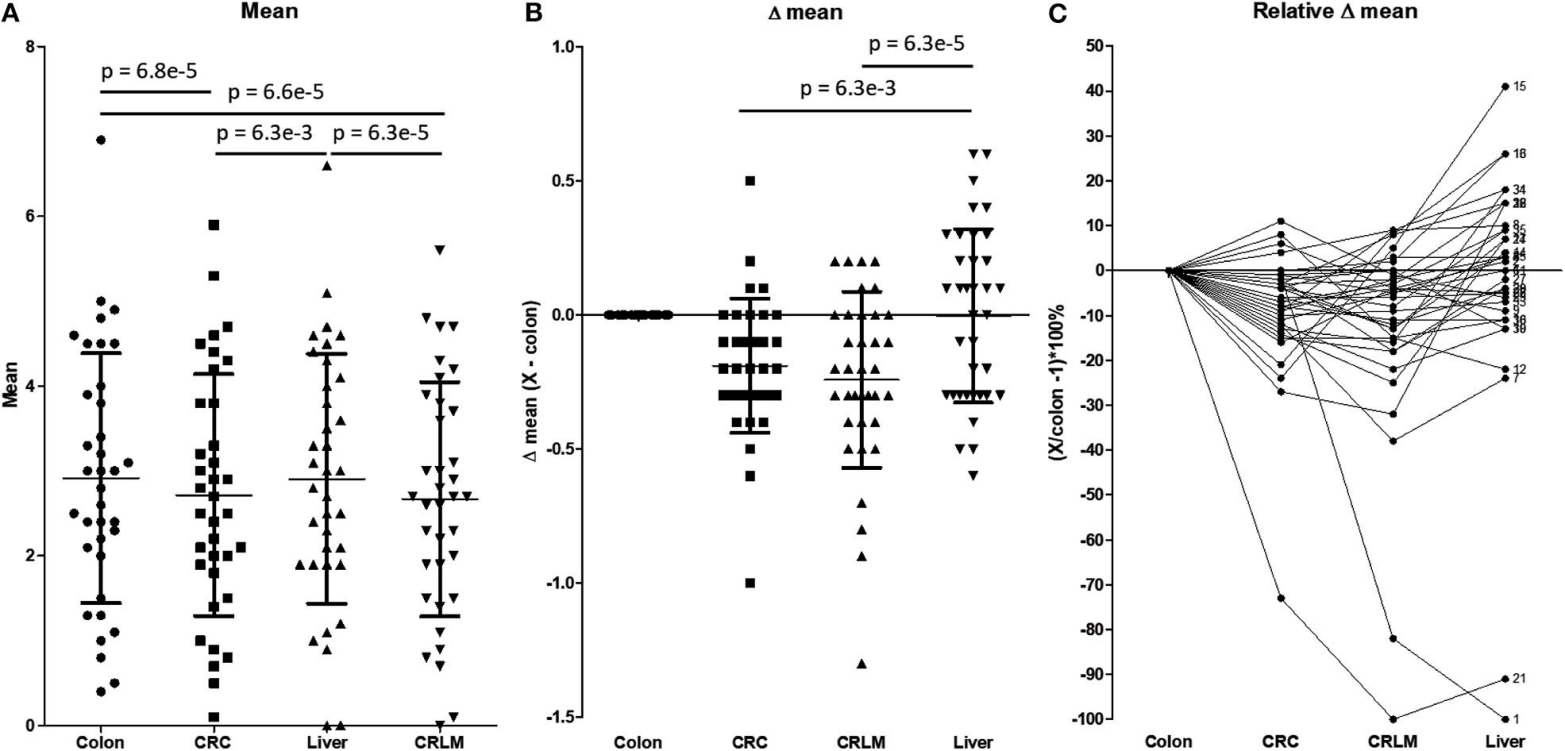
**(A)** The average number of hydroxylations per peptide. Tested for differences with a repeated paired *t*-test. *P*-values below 0.05/6 = 0.0083 are considered significant. **(B)** Absolute difference in the average number of hydroxylations per peptide with respect to colon tissue. *P*-values below 0.05/3 = 0.0167 are considered significant. **(C)** Relative difference in the number of hydroxylations with respect to colon tissue. The “X” in the legend of the Y-axis represents CRC, CRLM, or liver tissue, depending on the comparison made. Only significant values are presented as horizontal bars in **(A)** and **(B)**.

Figure 4 shows a more detailed analysis of the average number of hydroxylations. In Figure 4A, the average number of hydroxylations per peptide per tissue is shown. The overall average numbers of hydroxylations in CRC and CRLM are significantly lower than those in colon and liver tissue. Liver and colon tissue do not differ in number of hydroxylations, and also CRC and CRLM do not differ in this respect. Figure 4B shows the differences in the average number of hydroxylations in the comparison of colon tissue and the other tissues investigated. Figure 4B visualizes the overall shift in the average number of hydroxylations per peptide. The overall shift in the number of hydroxylations per peptide in liver tissue, with respect to colon tissue, does not significantly differ from zero. Overall, the mean of all average number of hydroxylations per peptide is significantly downregulated in CRC (*p* = 6.3^*^10^−3^) and in CRLM (*p* = 6.3^*^10^−5^) tissue compared to liver tissue. CRC and CRLM do not have a different shift in comparison to each other (*p* = 0.27). In Figure 4C, the relative differences in the average with respect to colon tissue are shown. There is a significant decrease in the average degree of hydroxylation, on average −9% in CRC (SD = 14%, *p* = 0.00056), and on average −12% in CRLM (SD = 22%, *p* = 0.0026) with respect to colon tissue, and liver tissue does not significantly differ from colon tissue (average = −3%, SD = 27%, *p* = 0.55).

Figure 4C visualizes how the degree of hydroxylation changes per peptide from colon to CRC, to CRLM, to liver tissue. For peptide 1, the average number of hydroxylations remains constant with the transition from colon to CRC tissue. However, in CRLM tissue the average number of hydroxylations decreases strongly. The degree of hydroxylation of peptide 1 in CRLM is similar to that in liver tissue. Regarding peptide 21, the average number of hydroxylations decreases strongly from colon to CRC, and from CRC to CRLM, and is fairly similar between CRLM and liver tissue. Regarding peptides 15 and 16, the average number of hydroxylations decreases from colon to CRC, and increases from CRC to CRLM to liver tissue. Peptides 1, 15, 16, and 21 appear to move toward liver tissue, when transitioning from colon, via CRC, to CRLM.

The largest number (*n* = 30) of different specific hydroxylation sites were detected between liver and colon tissue; a comparable number (*n* = 27) of different specific hydroxylation sites is present between colon and CRLM tissue. A closer study of specific hydroxylations that differ between liver and CRLM tissue, shows several unique differences, see Table 1. Peptide 7 with 0 hydroxylations is more prevalent in CRC and CRLM than in healthy colon and liver tissue. Peptide 13 with 1, peptide 15 with 1, peptide 16 with 1, peptide 18 with 0, peptide 19 with 3, and peptide 34 with 2 hydroxylations are abundantly present in colon, CRC and CRLM, but hardly in liver tissue. Peptide sequences are presented in the Supplementary S5, S6. Peptide 17 with 2, and peptide 27 with 5 hydroxylation are less abundant in CRLM compared to colon, CRC, and liver. Peptide 14 with 6, and peptide 24 with 5 hydroxylations, are present at similar levels in colon and liver, less in CRC, and the least in CRLM. The general pattern emerging from this data is consistent with a lower degree of hydroxylation in CRLM than in liver or colon tissue.

**Table 1:** Peptides with a specific hydroxylation form that are significantly different between liver and CRLM tissue, and the number of patients, per tissue, in whom the peptide was identified.

**Peptide**	**Number of prolines and lysines**	**Number of hydroxylations**	**14 Patients (matching colon, CRC, liver, and CRLM)**				**30 Patients (matching liver, and CRLM)**	
			**Colon**	**CRC**	**CRLM**	**Liver**	**CRLM**	**Liver**
7	7	0	0	2	8	0	15	0
13	4	1	14	14	12	3	26	13
14	8	6	13	7	3	14	5	27
15	5	1	14	14	12	3	26	6
16	9	2	13	13	8	1	20	3
17	4	2	14	10	4	11	10	18
18	4	0	7	9	11	1	26	3
19	6	3	8	11	11	0	22	3
24	9	5	5	3	0	8	2	13
27	9	5	13	12	3	9	5	17
34	9	2	12	11	10	0	20	2

### Urine Analysis

In urine, natural occurring peptides (NOPs) were identified that overlap with six peptides from 1 (21). Only for peptide 34, from 1, a NOP (AGPPGAPGAPGAPGPVGPAGKSGDRGETGP with 2 hydroxylations) was significantly different (*p-*value of 3.1^*^10^−4^) between urine from patients suffering from CRLM and healthy control urine. On the basis of the b- and y-ions from MS/MS spectra, the peptide AGPP(-OH)GAP(-OH)GAPGAPGPVGPAGKSGDRGETGP is identified to be hydroxylated at the 5th and 8th amino acid in colon tissue, CRC tissue, liver tissue, CRLM tissue, control urine, and CRLM urine.

## Discussion

The hydroxylation pattern of collagen alpha-1(I) is tissue-specific and each studied tissue has a unique hydroxylation pattern and a degree of hydroxylation as could be detected by PCA and statistical analysis. The variation in the hydroxylation pattern between liver and colon is larger than the variation within each tissue. Even though the average number of hydroxylations is comparable between colon and liver tissue, locally along the collagen alpha-1(I) chain the degree of hydroxylation is significantly different. The variation within a tissue and between healthy tissues implicates that every collagen triple helix has a unique hydroxylation pattern. Further studies are required to investigate whether prolines at specific sites are always hydroxylated. In addition, it would be of interest to ascertain whether a lack of specific hydroxyprolines could inhibit triple helix formation, or prevent incorporation of such a triple helix into supra-molecular structures. The hydroxylation pattern has a strong influence on the T_m_. We suggest that every triple helix consequently has a slightly different T_m_, and therefore interacts differently with the surroundings, and has different micro-unfoldings. In previous research it has been assumed that all prolines are hydroxylated; for example, to determine the T_m_ *in silico* (23). We show that this is not the case and that this assumption leads to an overestimation of the T_m_.

The collagen hydroxylation pattern is studied based on 36 selected peptides. Several of these selected peptides contain a missed cleavage present at arginine at lysine. A possible explanation is the presence of hydroxylation at the lysine moiety, which has been described to reduce the activity of trypsin (24). Although, peptide 1, which only contains one lysine moiety, is observed with and without a hydroxylation at lysine. This example shows that trypsin is able to cleave at a hydroxylated lysine. It might be more likely that the presence of aspartic acid and glutamic acid in close proximity to an arginine and lysine moiety reduces the efficiency of trypsin activity (25, 26).

We investigated if the hydroxylation pattern in a tumor migrates with the metastasis. The results of this study indicate that the hydroxylation degree in tumor tissue decreases significantly in comparison to degree of hydroxylation in healthy tissue. The degree of hydroxylation in CRC tissue is 9% lower and that in CRLM is 12% lower than in colon and liver tissue. Still, in the comparisons “colon vs. liver” and “CRLM vs. CRC” similar numbers of peptides were down- and upregulated. The hydroxylation pattern of several of the studied peptides appears to be tissue-dependent. For instance, peptide 1 was present in colon tissue with 0 and 1 hydroxylation, but in liver tissue only with 0 hydroxylation. CRLM tissue seems to be in an intermediate state, containing both peptide 1 with 0 hydroxylation and—to a lesser extent—peptide 1 with 1 hydroxylation. Peptide 1 contains just one lysine moiety, and zero proline moieties, further modification of 5-hydroxylysine (e.g., glycosylation) can prevent detection of peptide 1 with 1 hydroxylation. To our knowledge additional modifications of this specific lysine are not described in literature; nevertheless, if an additional modification is present, then this still indicates a significant difference between the tissues that have been compared. In line with the peptide 1 pattern, the hydroxylation degree of peptide 21 in colon tissue is similar to that in CRC tissue, and strongly reduced in both CRLM and liver tissue. The hydroxylation degree of peptides 15 and 16 decreases in CRC tissue, increases in CRLM tissue, and is even higher in liver tissue. Despite the reduced degree of hydroxylation in CRLM tissue, the general hydroxylation pattern does partly resemble liver tissue but also CRC, and colon.

A recently described method to detect CRLM is by measuring collagen natural occurring peptides (NOPs) in urine (27, 28). Higher sensitivity and specificity can potentially be obtained by adding distinctive collagen NOPs to the panel of molecular markers as described by us. In the present study, we found an overall reduction in the degree of collagen hydroxylation in tumor tissue, and identified specific peptides which are distinctive between CRLM and liver tissue. Comparison with previous data (21) showed that a NOP, which overlaps with peptide 34, was significantly different in urine of patients suffering from CRLM and in urine of healthy controls. This NOP is a promising candidate to improve the detection of CRLM in urine. Especially, because this peptide shows a biologically relation to the liver metastasis tumor. If the degree of collagen hydroxylation is too low, then the T_m_ of the formed triple helix can be too low and as a result this collagen will not be built into the supramolecular structures. Consequently we may find that there is a possibility to find NOPs in urine that have an even lower degree of hydroxylation than observed in this study, which could possibly be even more distinctive.

Hypoxia is a possible explanation of the overall decreased degree of hydroxylation in tumor tissue. McKeown clarified differences in the partial pressure of oxygen (pO_2_) in healthy and tumor tissue (29). The pO_2_ in rectal cancer tissue (30 mm Hg) is ~2 times lower than in healthy rectal tissue (52 mmHg); the pO_2_ in liver tumor (6.0 mmHg) is ~5 times lower than in healthy liver tissue (30 mmHg). Utting et al. (16) showed that hypoxia reduces the degree of hydroxylation and has the largest effect on Hyl formation, and a relative small effect on Hyp formation. In contrast to the above reasoning, the hydroxylation degree of peptides 1 and 21 is decreased in liver tissue in comparison to colon tissue. Peptide 15 and 16, also in contrast to the overall trend, have an increasing hydroxylation degree from CRC to CRLM to liver tissue. Hypoxia can explain the general trend observed, although specific cases such as peptides 1, 15, 16, and 21 do not agree with these observations of McKeown en Utting et al. (16) and may be explained by unknown mechanisms by which hydroxylation is regulated specifically.

Proline and lysine can be enzymatically hydroxylated. Van Huizen et al. (20) described the general upregulation of enzymes related to collagen production and hydroxylation in CRLM. Except the specific enzyme P4HB, the beta unit of 4-prolyl hydroxylase, is strongly reduced in CRLM (11, SD = 5 unique peptides) compared to liver tissue (20, SD = 5 unique peptides) (fold change = 0.48, *p* = 1.6^*^10^−18^). P4HB is crucial for the solubility and folding of 4-prolyl hydroxylase. In the present study in colon tissue, on average, 12.8 peptides (SD = 4.1) belonging to P4HB were identified, and in CRC on average 16.1 peptides (SD = 3.9). The downregulation of P4HB in CRLM in comparison to liver tissue might explain the decreased degree of hydroxylation; in CRC tissue, however, P4HB is higher than in colon tissue while the hydroxylation degree is also decreased.

The extracellular matrix is involved in tumor development (30). It has been proposed that the extracellular matrix can act as a tumor suppressor as long as it remains in its'natural shape'; if the “natural morphology” is lost, then it could become a tumor promoter (30). In this study we have shown that besides the collagen expression, also the collagen hydroxylation is significantly different between tumor and normal tissue. Further research is required to understand the effect of the reduced degree of collagen hydroxylation on tumor proliferation and to identify the mechanisms that are involved in the regulation of changed hydroxylation levels in cancer.

## Data Availability Statement

The datasets presented in this study can be found in online repositories. The names of the repository/repositories and accession number(s) can be found at: PRIDE database (PXD015015).

## Ethics Statement

The studies involving human participants were reviewed and approved by the MERC (medical ethical review committee), Erasmus MC. Written informed consent from the patients was not required to participate in this study in accordance with the national legislation and the institutional requirements.

## Author Contributions

NH, PB, JI, and TL designed research. NH, PB, and MD performed research. NH and JR contributed to new analytical tools. NH and PB analyzed data. All authors were involved in writing the paper.

## Conflict of Interest

The authors declare that the research was conducted in the absence of any commercial or financial relationships that could be construed as a potential conflict of interest.

## Abbreviations

3Hyp: 3-hydroxyproline
4Hyp: 4-hydroxyproline
4xHyp: 4-hydroxyproline at the Xaa-position
CRC: Colorectal cancer
CRLM: Colorectal liver metastasis
Hyp: Hydroxyproline
IQR: Interquartile range
NOP: Natural occurring peptides
pO_2_: Partial pressure of oxygen
T_m_: Melting temperature of collagen.
