# Supplementary material for: Down-Regulation of Collagen Hydroxylation in Colorectal Liver Metastasis
Source: Front Oncol. 2020 Sep 30;10:557737. doi: 10.3389/fonc.2020.557737 (PMC7561380; doi:10.3389/fonc.2020.557737)
Supplement: Supplementary file 3 [file Data_Sheet_2.docx]

**Supplemental S-2, The R-code used to perform the PCA analysis**

#based on http://www.sthda.com/english/articles/31-principal-component-methods-in-r-practical-guide/118-principal-component-analysis-in-r-prcomp-vs-princomp/

#install.packages

install.packages("factoextra")

install.packages("PerformanceAnalytics")

library("PerformanceAnalytics")

library(factoextra)

library(car)

library(rgl)

library(ggplot2)

library(RColorBrewer)

#data

data <- read.table(file = "Data location", header = TRUE, sep = "\t", as.is = TRUE, row.names=1)

#1:30 Liver

#31:60 CRLM

#61:74 colon

#75:88 CRC

groups <- c(rep("liver",30),rep("CRLM",30),rep("colon",14),rep("CRC",14)); data_red <- data

groups <- c(rep("colon",14),rep("CRC",14)); data_red <- data[c(61:88),]

groups <- c(rep("Liver",30),rep("CRLM",30)); data_red <- data[c(1:60),]

groups <- c(rep("CRLM",30),rep("CRC",14)); data_red <- data[c(31:60,75:88),]

groups <- c(rep("liver",30),rep("Colon",14)); data_red <- data[c(1:30,61:74),]

groups <- c(rep("CRLM",30), rep("CRC",14)); data_red <- data[c(31:60,75:88),]

groups <- c(rep("liver",30)); data_red <- data[c(1:30),]

groups <- c(rep("CRLM",30)); data_red <- data[c(31:60),]

groups <- c(rep("Colon",14)); data_red <- data[c(61:74),]

groups <- c(rep("CRC",14)); data_red <- data[c(75:88),]

#14 vs 14 matched

data <- read.table(file = "Data location", header = TRUE, sep = "\t", as.is = TRUE, row.names=1)

#1:14 Liver

#15:28 CRLM

#29:42 colon

#43:56 CRC

groups <- c(rep("liver",14),rep("CRLM",14),rep("colon",14),rep("CRC",14)); data_red <- data

groups <- c(rep("colon",14),rep("CRC",14)); data_red <- data[c(29:56),]

groups <- c(rep("Liver",14),rep("CRLM",14)); data_red <- data[c(1:28),]

groups <- c(rep("CRLM",14),rep("CRC",14)); data_red <- data[c(15:28,43:56),]

groups <- c(rep("liver",14),rep("Colon",14)); data_red <- data[c(1:14,29:42),]

groups <- c(rep("CRLM",14), rep("CRC",14)); data_red <- data[c(15:28,43:56),]

groups <- c(rep("colon",14), rep("CRLM",14)); data_red <- data[c(29:42,15:28),]

#Preparing data

data_red <- data_red[,colSums(data_red != 0) > 0] #remove columns that are empty.

data_red.pca <- prcomp(data_red, center = TRUE) #no scale. = TRUE, data is 0 or 1

#PCA results

summary(data_red.pca)

fviz_eig(data_red.pca)

fviz_contrib(data_red.pca, choice="var", axes =1:2, invisible = "labels") #plots contribution of each protein to the PCA model axes = ? tells to which dimension

fviz_contrib(data_red.pca, choice="var", axes =1:2, top=25) + #plots contribution of each protein to the PCA model axes = ? tells to which dimension

theme(axis.text.x = element_text(angle = 90))

#contribution of var

fviz_pca_var(data_red.pca,

label="none",

geom = "point",

col.var="contrib")+

scale_color_gradient2(low="white", mid="blue",high="red", midpoint=1.5)

#PCA

fviz_pca_ind(data_red.pca,

repel=TRUE,

mean.point=FALSE,

habillage = groups,

addEllipses = TRUE,

title = "Hydrox variation",

label = "none",

pointsize = 2)

#PCA w/o groups, with labels

fviz_pca_ind(data_red.pca,

repel=TRUE,

invisible = "quali",

addEllipses = TRUE,

title = "Hydrox variation")

#3d PCA

fviz_contrib(data_red.pca, choice="var", axes =1:3, top=25) + #plots contribution of each protein to the PCA model axes = ? tells to which dimension

theme(axis.text.x = element_text(angle = 90))

data_red.3dpca <- get_pca_ind(data_red.pca)$coord

x <- data_red.3dpca[,1]

y <- data_red.3dpca[,2]

z <- data_red.3dpca[,3]

scatter3d(x,y,z,

groups = as.factor(groups),

surface.col = c("purple", "blue", "green", "orange"),

surface = FALSE,

ellipsoid = TRUE,

grid=TRUE,

xlab = "dim.1", ylab= "dim.2", zlab="dim.3")

rgl.viewpoint(0, 20)

for( i in seq(1,720, by=1.5)){

rgl.viewpoint(i,i*0.04)

}

#Contribution of in the individuals

fviz_pca_ind(data_red.pca,

repel=TRUE,

col.ind="cos2",

title = "Hydrox variation",

label = "none")+

scale_color_gradient2(low="white", mid="blue",high="red", midpoint=0.3)

#Mean relation

cor_m <- read.table(file ="clipboard", sep="\t", header = TRUE)

chart.Correlation(cor_m, histogram = TRUE, pch=19)

plot(cor_m[,1], cor_m[,2], col = "green")

points(cor_m[,1], cor_m[,3], col = "blue")

points(cor_m[,1], cor_m[,4], col = "red")

hist_m <- read.table(file = "clipboard", sep ="\t", header = TRUE)

hist(hist_m[,1], col = rgb(1,0,0,0.5), ylim = c(0,1), freq = F)$count

hist(hist_m[,2], col = rgb(0,1,0,0.5), add = T, freq = F)$count

hist(hist_m[,3], col = rgb(0,0,1,0.5), add = T, freq = F)$count

hist(hist_m[,4], col = rgb(1,1,0,0.5), add = T, freq = F)$count

#Pretty figure of PCA result

#Liver/CRLM data

age <- c(74,74,62,68,65,77,74,76,43,86,73,73,76,60,64,70,36,77,49,73,71,66,61,58,64,69,78,54,78,59)

gender <- c("M","M","M","F","M","M","F","M","M","M","M","M","F","M","M","F","M","M","F","F","M","M","F","M","F","M","M","F","F","M")

location <- c("Sigmoïd","Sigmoïd" ,"Rectum" ,"Rectum" ,"Rectum" ,"Rectum" ,"Rectum" ,"Colon descendant" ,

"Rectum" ,"Rectum" ,"Colon ascendant" ,"Colon ascendant" ,"Sigmoïd" ,"Rectum" ,"Rectum" ,

"Sigmoïd" ,"Sigmoïd" ,"Rectum" ,"Rectum" ,"Rectum" ,"Rectum" ,"Sigmoïd" ,"Sigmoïd" ,"Sigmoïd" ,

"Colon descendant" ,"Sigmoïd" ,"Rectum" ,"Rectum" ,"Rectum" ,"Sigmoïd")

#Colon/CRC data

age <- c(61, 52, 44, 63, 75, 56, 61, 84, 74, 74, 64, 69, 69, 43)

gender <- c("M", "F", "F", "M", "M", "M", "M", "M", "F", "F", "M", "F", "M", "M")

location <- c("Rectum","Rectum" ,"Rectum" ,"Rectum" ,"Rectum" ,"Sigmoïd" ,"Rectum" ,"Rectum" ,"Sigmoïd" ,"Rectum" ,"Sigmoïd" ,"Sigmoïd" ,"Sigmoïd" ,"Rectum")

#x,y originate from 3d PCA section

dataGG <- data.frame(x,y,location,gender,age) #Tumor data frame is required for ggplot

dataGG <- data.frame(x,y,gender,age) #Healthy data frame is required for ggplot

#healthy

ggplot(data = dataGG, aes(x = x, y = y)) +

geom_point(aes(col = age, size = gender)) +

scale_size_manual(values = c(3,6)) +

scale_color_continuous(low = "yellow", high = "red") +

xlim(-4,4) + ylim(-3,3)

#tumor

ggplot(data = dataGG, aes(x = x, y = y)) +

geom_point(aes(col = age, size = gender, shape = location)) + #remove location

scale_size_manual(values = c(3,6)) +

scale_color_continuous(low = "yellow", high = "red") +

xlim(-2,6) + ylim(-2,3)
