# Supplementary material for: Down-Regulation of Collagen Hydroxylation in Colorectal Liver Metastasis
Source: Front Oncol. 2020 Sep 30;10:557737. doi: 10.3389/fonc.2020.557737 (PMC7561380; doi:10.3389/fonc.2020.557737)
Supplement: Supplementary file 4 [file Data_Sheet_3.docx]

**Supplemental S-3, The R-script used to the statistical analysis**

#library

library(abind)

library(beepr); beep(sound=4)

#import data

#selected from #2, w/o C or M, at least 20 times in liver/CRLM, in all groups.

peptide <- read.table(file = " File with peptides", sep="\t",header = FALSE,as.is = TRUE)

#Liver vs CRLM 30 matched

pat <- read.csv(file = "Data location" ,sep="\t",header = TRUE ,as.is = TRUE)

group1 <- read.csv(file = "Data location" ,sep="\t",header = FALSE,as.is = TRUE)

group2 <- read.csv(file = "Data location" ,sep="\t",header = FALSE,as.is = TRUE)

#Liver vs CRLM 14 matched

pat <- read.csv(file = "Data location" ,sep="\t",header = TRUE ,as.is = TRUE)

group1 <- read.csv(file = "Data location" ,sep="\t",header = FALSE,as.is = TRUE)

group2 <- read.csv(file = "Data location" ,sep="\t",header = FALSE,as.is = TRUE)

#colon data

pat <- read.csv(file = "Data location" ,sep="\t",header = TRUE ,as.is = TRUE)

group1 <- read.csv(file = "Data location" ,sep="\t",header = FALSE,as.is = TRUE)

group2 <- read.csv(file = "Data location" ,sep="\t",header = FALSE,as.is = TRUE)

#Liver vs colon

pat <- read.csv(file = "Data location" ,sep="\t",header = TRUE ,as.is = TRUE)

group1 <- read.csv(file = "Data location" ,sep="\t",header = FALSE,as.is = TRUE)

group2 <- read.csv(file = "Data location" ,sep="\t",header = FALSE,as.is = TRUE)

#CRLM vs CRC

pat <- read.csv(file = "Data location" ,sep="\t",header = TRUE ,as.is = TRUE)

group1 <- read.csv(file = "Data location" ,sep="\t",header = FALSE,as.is = TRUE)

group2 <- read.csv(file = "Data location" ,sep="\t",header = FALSE,as.is = TRUE)

#Colon vs CRLM

pat <- read.csv(file = "Data location" ,sep="\t",header = TRUE ,as.is = TRUE)

group1 <- read.csv(file = "Data location" ,sep="\t",header = FALSE,as.is = TRUE)

group2 <- read.csv(file = "Data location" ,sep="\t",header = FALSE,as.is = TRUE)

#numb of patiets and numb of peptides

patn <- length(pat[,1])

pepn <- length(peptide[,1])

#____________________cube1____________________

#Creating data cube

cube1 <-array(dim=c(14,(pepn+1),(patn+1)))

cube1[2:14,1,1:(patn+1)] <- c(seq(0,10,by=1),"freq","mean") #x-axis hydroxylation 0:7

cube1[1,2:(pepn+1),1:(patn+1)] <- peptide[1:pepn,1] #y-axis peptides

cube1[1,1,1:(patn+1)] <- c("overal",pat[1:patn,1]) #z-axis patient name

pb<- txtProgressBar(min=2,max = pepn+1, style = 3) #create progress bar

#Fill in data cube

for(y in 2:(pepn+1)){

setTxtProgressBar(pb, y) #create progress bar

for(x in 2:12){

for(z in 2:(patn+1)){

comb <- paste(cube1[1,1,z],cube1[1,y,1],cube1[x,1,1])

comb <- gsub("\\s", "-",comb)

for(g in 1: length(group1[,1])){

if(group1[g,1] == comb){

cube1[x,y,z] <- as.numeric(x-2) #x is position in cube, x-2, is number of hydroxylations

}}}} }

close(pb) #create progress bar

#Count to create overal

for(x in 2:12){

cube1[x,2:(pepn+1),1] <- (patn)-rowSums(is.na(cube1[x,2:(pepn+1),2:(patn+1)]))

}

#count to create freq and mean

for(z in 2:(patn+1)){

cube1[13,2:(pepn+1),z] <- 11-colSums(is.na(cube1[2:12,2:(pepn+1),z])) #freq

for(y in 2:(pepn+1)){

cube1[14,y,z] <- mean(as.numeric(cube1[2:12,y,z]),na.rm=TRUE) #mean

}}

#__________________________________cube2______

#Creating data cube

cube2 <-array(dim=c(14,(pepn+1),(patn+1)))

cube2[2:14,1,1:(patn+1)] <- c(seq(0,10,by=1),"freq","mean") #x-axis hydroxylation 0:7

cube2[1,2:(pepn+1),1:(patn+1)] <- peptide[1:pepn,1] #y-axis peptides

cube2[1,1,1:(patn+1)] <- c("overal",pat[1:patn,2]) #z-axis patient name

pb<- txtProgressBar(min=2,max = pepn+1, style = 3) #create progress bar

#Fill in data cube

for(y in 2:(pepn+1)){

setTxtProgressBar(pb, y) #create progress bar

for(x in 2:12){

for(z in 2:(patn+1)){

comb <- paste(cube2[1,1,z],cube2[1,y,1],cube2[x,1,1])

comb <- gsub("\\s", "-",comb)

for(g in 1: length(group2[,1])){

if(group2[g,1] == comb){

cube2[x,y,z] <- as.numeric(x-2) #x is position in cube, x-2, is number of hydroxylations

}}}} }

close(pb) #create progress bar

#Count to create overal

for(x in 2:12){

cube2[x,2:(pepn+1),1] <- (patn)-rowSums(is.na(cube2[x,2:(pepn+1),2:(patn+1)]))

}

#count to create freq and mean

for(z in 2:(patn+1)){

cube2[13,2:(pepn+1),z] <- 11-colSums(is.na(cube2[2:12,2:(pepn+1),z])) #freq

for(y in 2:(pepn+1)){

cube2[14,y,z] <- mean(as.numeric(cube2[2:12,y,z]),na.rm=TRUE) #mean

}}

#_________________cube_comparison______________

cube_compare <- function(cube1,cube2,patn,pepn){

wil <- array(dim=c(pepn,11)) #array to safe this data in

for(y in 2:(pepn+1)){

#frequency of hydroxylation

wil[y-1,1] <- wilcox.test(as.numeric(cube1[13,y,2:(patn+1)]),as.numeric(cube2[13,y,2:(patn+1)]),paired = TRUE, alternative = "two.sided")$p.value

if (is.na(wil[y-1,1])){

wil[y-1,1] = 1

}

wil[y-1,2] <- mean(as.numeric(cube1[13,y,2:(patn+1)]))

wil[y-1,3] <- mean(as.numeric(cube2[13,y,2:(patn+1)]))

wil[y-1,4] <- sum(as.numeric(cube1[13,y,2:(patn+1)])<1)

wil[y-1,5] <- sum(as.numeric(cube2[13,y,2:(patn+1)])<1)

#mean number of hydroxylations

wil[y-1,6] <- wilcox.test(as.numeric(cube1[14,y,2:(patn+1)]),as.numeric(cube2[14,y,2:(patn+1)]),paired = TRUE, alternative = "two.sided",na.action=na.omit)$p.value

if (is.na(wil[y-1,6])){

wil[y-1,6] = 1

}

wil[y-1,7] <- mean(as.numeric(cube1[14,y,2:(patn+1)]), na.rm = TRUE)

wil[y-1,8] <- mean(as.numeric(cube2[14,y,2:(patn+1)]), na.rm = TRUE)

p = 0.05/pepn

if(wil[y-1,1] <= p){

wil[y-1,9] = 1 #overal

wil[y-1,10] = 1 #mean freq

}else{wil[y-1,10] = 0} #mean freq

if(wil[y-1,6] <= p){

wil[y-1,9] = 1 #overal

wil[y-1,11] = 1 #meanspecific

}else{wil[y-1,11] = 0} #mean specific

if(is.na(wil[y-1,9])){wil[y-1,9] = 0} #fillup overal with 0 if neither mean nor freq is different

}

return(wil)

} #function to do cube comparison

#per peptide level the freq and mean are extracted and compared between the cubes

wil <- cube_compare(cube1,cube2,patn,pepn)

par(mfrow=c(2,2))

plot(wil[,2]-wil[,3],wil[,7]-wil[,8],

main = "overal freq or mean p<0.05",

xlim = c(-1.5,1.5), ylim=c(-1.5,1.5),

xlab="freq (normal-tumor)",ylab="mean (normal-tumor)",

col= ifelse(wil[,9] == 1, "red","black"),

pch=19)

abline(h=0)

abline(v=0)

y <- wil[,2]-wil[,3]

x <- wil[,7]-wil[,8]

text(x~y, labels = ifelse(wil[,9] == 1,seq(1,pepn,by=1), ""), pos=4)

plot(wil[,2]-wil[,3],wil[,7]-wil[,8],

main = "freq p<0.05",

xlim = c(-1.5,1.5), ylim=c(-1.5,1.5),

xlab="freq (normal-tumor)",ylab="mean (normal-tumor)",

col= ifelse(wil[,10] == 1, "red","black"),

pch=19)

abline(h=0)

abline(v=0)

y <- wil[,2]-wil[,3]

x <- wil[,7]-wil[,8]

text(x~y, labels = ifelse(wil[,10] == 1,seq(1,pepn,by=1), ""), pos=4)

plot(wil[,2]-wil[,3],wil[,7]-wil[,8],

main = "mean p<0.05",

xlim = c(-1.5,1.5), ylim=c(-1.5,1.5),

xlab="freq (normal-tumor)",ylab="mean (normal-tumor)",

col= ifelse(wil[,11] == 1, "red","black"),

pch=19)

abline(h=0)

abline(v=0)

y <- wil[,2]-wil[,3]

x <- wil[,7]-wil[,8]

text(x~y, labels = ifelse(wil[,11] == 1,seq(1,pepn,by=1), ""), pos=4)

#_____Individual_hydroxylation_position_comparison__

#Paired data will be analysed with McNemar and unpaired with Chi-Square.

mcnemar <- function(cube1,cube2,patn,pepn){

result <- array(dim=c(12,(pepn+1)))

result[1,2:(pepn+1)] <- peptide[,1]

result[2:12,1] <- seq(0,10,by=1)

pp <- 0; pn <- 0; np <- 0; nn <- 0

#McNemar

for(y in 2:(pepn+1)){

for(x in 2:12){

pp <- 0; pn <- 0; np <- 0; nn <- 0 #1 group1, 2 group 2 n = negative, p = positive, counters to construct matrix table

for(z in 2:patn){

if( is.na(cube1[x,y,z]) == "FALSE" && is.na(cube2[x,y,z]) == "FALSE"){

pp <- pp + 1

}else if(is.na(cube1[x,y,z]) == "FALSE" && is.na(cube2[x,y,z]) == "TRUE" ){

pn <- pn + 1

}else if(is.na(cube1[x,y,z]) == "TRUE" && is.na(cube2[x,y,z]) == "FALSE"){

np <- np + 1

}else if(is.na(cube1[x,y,z]) == "TRUE" && is.na(cube2[x,y,z]) == "TRUE"){

nn <- nn + 1

} }

m <- matrix(c(pp,pn, np, nn),2)

result[x,y] <- mcnemar.test(m)$p.value

}}

return(result)

}

result <- mcnemar(cube1,cube2,patn,pepn)

#_______________Permutation____________________

#create cube1 and cube2 with the data of interest.

#number of permutations

n_perm = 100000

#cube 3 = cube1 + cube2

cube3 <- abind(cube1[,,2:(patn+1)], cube2[,,2:(patn+1)])

#cube3 <- cube1[,,2:(patn+1)] #adapt within liver, only 1 cube is available, use "liver data"

#cube3 <- cube2[,,2:(patn+1)] #adapt within CRLM, only 1 cube is available, use"liver data"

cube4 <- array(dim=c(14,(pepn+1),(patn)))

cube5 <- array(dim=c(14,(pepn+1),(patn)))

#Calculate number of mcnemar calculations

total <- 0

for(x in 2:12){

for(y in 2:37){

if(sum(!is.na(cube3[x,y,])) >0){

total <- total + 1

}}}

perm_result <- array(dim=c(n_perm,6))

colnames(perm_result) <- c("freq hint", "freq sign", "mean hint", "mean sign", "hydrox hint", "hydrox sign")

for(i in 1:n_perm){

rand <- sample(1:30, replace = FALSE) #create random numbers to redevide data, adapt within liver 1:30

cube4 <- cube3[,,rand[1:15]] #adapt within liver 1:15

cube5 <- cube3[,,rand[16:30]] #adapt within liver 16:30

comp <- cube_compare(cube4,cube5,(patn/2-1),pepn)#adapt within liver patn/2 (dont remove -1) (1 group split in 2)

hydr <- mcnemar(cube4,cube5,patn/2,pepn) #adapt within liver patn/2 (1 group split in 2)

#write results

perm_result[i,1] <- sum(comp[,1]<0.05)

perm_result[i,2] <- sum(comp[,1]<(0.05/pepn))

perm_result[i,3] <- sum(comp[,6]<0.05)

perm_result[i,4] <- sum(comp[,6]<(0.05/pepn))

perm_result[i,5] <- sum(hydr[2:12,2:37]<0.05, na.rm = TRUE)

perm_result[i,6] <- sum(hydr[2:12,2:37]<(0.05/total), na.rm = TRUE)

print(i)

}
